# Mobility dynamics of migrant workers and their socio-behavioral parameters related to malaria in Tier II, Artemisinin Resistance Containment Zone, Myanmar

**DOI:** 10.1186/s12889-015-2241-0

**Published:** 2015-09-14

**Authors:** Thaung Hlaing, Khin Thet Wai, Tin Oo, Nyan Sint, Tun Min, Shwe Myar, Khin Nan Lon, Myo Myint Naing, Tet Toe Tun, Nay Lin Yin Maung, Gawrie N. L. Galappaththy, Krongthong Thimarsan, Tin Tin Wai, Lwin Ni Ni Thaung

**Affiliations:** Department of Public Health, Naypyitaw, Myanmar; Department of Medical Research, No. 5 Ziwaka Road, Dagon Township, Yangon, 11191 Myanmar; Malaria Unit, WHO Country Office, Yangon, Myanmar

## Abstract

**Background:**

Areas with dynamic population movements are likely to be associated with higher levels of drug-resistant malaria. Myanmar Artemisinin Resistance Containment (MARC) Project has been launching since 2012. One of its components includes enhancing strategic approaches for mobile/migrant populations. We aimed to ascertain the estimated population of mobile migrant workers and their families in terms of stability in work setting in townships classified as tier II (areas with significant inflows of people from areas with credible evidence of artemisinin resistance) for Artemisinin resistance; to identify knowledge, attitudes and practices related to prevention and control of malaria and to recommend cost-effective strategies in planning for prevention and control of malaria.

**Methods:**

A prospective cross-sectional study conducted between June to December 2013 that covered 1,899 migrant groups from 16 tier II townships of Bago Region, and Kayin and Kayah States. Trained data collectors used a pre-tested and subsequently modified questionnaire and interviewed 2,381 respondents. Data of migrant groups were analyzed and compared by category depending upon the stability of their work setting.

**Results:**

The estimated population of the 1,899 migrant groups categorized into three on the nature of their work setting was 56,030. Bago region was the commonest reported source of origin of migrant groups as well as their transit. Malaria volunteers were mostly within the reach of category 1 migrant groups (43/66, 65.2 %). Less stable migrant groups in category 3 had limited access to malaria information (14.7 %) and malaria care providers (22.1 %), low level of awareness and use of long-lasting insecticide-treated nets (46.6 and 38.8 %). Also, they had poor knowledge on malaria prevention on confirming suspected malaria and on using artemisinin combined therapy (ACT). Within two weeks prior to the survey, only 16.5 % of respondents in all categories combined reported acute undifferentiated fever.

**Discussion and Conclusions:**

Mobility dynamics of migrant groups was complex and increased their vulnerability to malaria. This phenomenon was accentuated in less stable areas. Even though migrant workers were familiar with rapid diagnostic tests for malaria, ACT still needed wide recognition to improve practices supportive of MARC including the use of appropriate personal protection. High mobility calls for re-designation of tier II townships to optimize ACT resistance containment.

**Electronic supplementary material:**

The online version of this article (doi:10.1186/s12889-015-2241-0) contains supplementary material, which is available to authorized users.

## Background

Migration process encompasses any movement of people, whatever its length, composition and causes. It includes migration of refugees, displaced people, uprooted people and economic migrants [1]. The magnitude of population migration has been increasing as reflected by global and regional trends and patterns of mobility. It is obvious that there are links between population migration, work patterns and malaria transmission globally and in the Greater Mekong sub-region including in Myanmar [2–4]. Human population movement patterns and the risk of malaria vary substantially across spatial and temporal scales, socio-demographic sub-groups, and motivation for travel, so multiple data sets are likely required for quantification of movement [5]. Higher levels of drug-resistant malaria are also reported to from locations with more dynamic population movements than those with less movement. The significant impact of migration on patterns of malaria infection and disease with implications for malaria control has been reported [6, 7].

In Myanmar, both malaria-related morbidity and mortality have been decreasing in recent years in the areas of high endemicity. However, the problem of drug resistance has been emerging especially in the southern part of Myanmar which borders Thailand [8]. This problem is likely to be associated with population mobility. In line with the National Malaria Control Policy, Myanmar Artemisinin Resistance Containment (MARC) Project has been launching in 2012, and one of its components includes introducing strategic approaches for mobile/migrant populations. Various types of organizations partner the National Malaria Control Program (NMCP) in this connection [2, 9]. Opportunities and challenges emerge in prevention and control of malaria in mobile and migrant populations in MARC zone including channeling malaria-related information.

Special aspects that make migrants at-risk of malaria include their socio-cultural context, economic activities, accessibility to health services, access to antimalarials, service availability and readiness, behavioral parameters, language barriers, emergence of drug-resistant strains, activities for social mobilization and ecology. Very few studies in Myanmar highlighted those issues at vulnerable sites [10, 11] that may need effective diagnostic, therapeutic and preventive measures. There were certain limitations of a previous study that covered 19 townships of Mon State, Tanintharyi Region and Bago Region from tier I (areas with credible evidence of artemisinin resistance) of MARC zone in 2012[12]. That particular survey did not provide socio-behavioral data and had a lack of clarity in defining migrants within the context of prevention and control of malaria in MARC project. Therefore, this present research work aimed to a) ascertain he estimated population of mobile migrant workers and their families in terms of stability in work setting in tier II townships (areas with significant inflows of people from tier I); b) to identify the knowledge, attitudes and practices related to prevention and control of malaria by category of migrant groups and c) to recommend cost-effective strategies in planning for prevention and control of malaria in mobile/migrant populations in tier II townships, MARC zone, Myanmar.

## Methods

### Study design

The prospective cross-sectional study was conducted from September to December 2013.

### Study sites and population

Altogether 16 townships in tier II, MARC zone in Bago Region (*n* = 10), Kayin State (*n* = 3), and Kayah State (*n* = 3) were included in the study. The study population comprised hard-to-reach groups of varying types of economic activity and mobility patterns ranging from seasonal to a long stay in the study areas. Migrant in this study was defined as a person who resided or remained in another place for an extended period, including seasonal migrants. Likewise, the mobile population covered any person who moves from one area to another either internally or externally, usually for a short period (less than one month). As regards the setting of specific migrant groups, *category 1* referred to permanent or semi-permanent work settings with high social capital, where sustainable results can be achieved for malaria control; *Category 2*: semi-permanent work settings with moderate social capital, where sustainable community-based results can be achieved for malaria control; *Category 3*: small, often temporary work sites, with low social capital and resource availability. This classification was arbitrary prior to the actual survey as a result of discussions between authorities from the National Malaria Control Program (NMCP), WHO Country Office and Department of Medical Research. Permanent or semi-permanent work settings referred to registered work-sites (large and medium size companies) hiring migrant workers. These work settings usually had office structures, a regular system of paying work charges, and easy means of communication. In contrast, the temporary work setting covered those without any structure and moving around for better economic opportunities. This classification did not depend upon the income/assets in specific migrant groups.

### Sampling and sample size

The multi-stage sampling method was employed. Firstly, the NMCP used purposive sampling to select the study townships (*n* = 16) in tier II, MARC zone. Then, the consultant malariologist concerned of the specific region/state selected the mobile/migrant groups (*n* = 1,899). The number of migrant groups selected in each township was unequal due to feasibility and density. However, the ratio of at least 1:2 was considered in selecting migrant groups stratified as categories 1 and 2 (more stable and permanent work settings) versus category 3 (less stable work settings). The ratio of 1:2 was an approximate number deriving from the available numbers of registered work sites. Those sites included rubber plantations, gold mines compared to unregistered and temporary work settings such as paddy farms, road building, etc. The assumption was based on the real-time feedback and the recent experience of the regional malaria officers. They were familiar with different categories of migrant groups engaged in different types of work settings in their region as they already conducted the distribution of LLIN to those groups. The respondent-driven sampling procedure could not be used due to time and funding restraints although it was most appropriate for hard-to-reach groups. The KAP questionnaire survey covered 2,381 mobile/migrants ranging from 99–294 respondents selected consecutively per study site. The sample size per study site of around 100 met the assumptions of 10 % incorrect practices related to malaria in tier II, MARC zone at 95 % confidence level and the precision of 6 %.

### Data collection

Data collection tools from the most recent completed survey of mobile/migrant workers in tier I townships were thoroughly discussed among the researchers from the Department of Medical Research (Lower Myanmar) (DMR-LM) and personnel from the NMCP and modified accordingly in late June 2013. The team from DMR (LM) trained 42 data collectors from four States/Regions in September 2013. Two sets of data were available from interviews by trained data collectors using the pre-tested and modified questionnaire to cover the information of mobile/migrants. For the first data set on migrant aggregates, the specific form was used to interview 2–3 representatives from each migrant group. The form included the category of migrant group, patterns of mobility (source of origin, transit, duration of stay, and place for next move), nature of work within the context of malaria transmission, estimated population structure specific by age and sex, pregnant women, estimated number of living arrangements, and access to health care facilities and malaria care providers. The second data set covered the individual structured interview form with 44 items in three sections: section A-social and demographic information (15 items), section B-bed nets (12 items), and section C- malaria information (17 items) (Additional file 1).

### Data analysis

The team from the DMR-LM trained data operators from 3 States/Regions for data entry by using EPI DATA version 3.0 in December 2013. Data were checked for errors and inconsistencies and analyzed by SPSS version 17.0. Frequency distributions and cross-tabulations of variables of interest were done, and data were described by univariate measures. Crude odds ratios and 95 % confidence intervals were computed as necessary. Chi-squared test was used to test the significance. Data of migrant groups were analyzed and compared by category at two levels: aggregate (group) level and the individual level.

### Ethical considerations

This study is a continuation of a study entitled “Diversities of mobile populations in the context of strengthening collaboration for malaria interventions: The study was approved by the Ethics Review Committee of DMR-LM. Privacy, anonymity and confidentiality issues were observed during data collection following an informed consent.

## Results

For the group/aggregate level data, those sites surveyed in Kayah State, Kayin State, and Bago Region were classified into categories 1, 2 and 3. The total number of migrant groups varied by townships that ranged from 7 in Kayah State to 386 in Bago Region. Among 1,899 migrant groups, 94 (4.9 %) belonged to category 1 followed by 392 groups in category 2 (20.6 %) and the majority were from category 3(1413/1899; 74.4 %).

Across three categories of migrant groups, the estimated total population in category 1 was less than 9,000, followed by category 2 that covered over 20,000. In category 3, there were over 36,000 migrants (Table 1). Female to male ratio of all age structure in the combined sample was 1: 1.3 (28,608 female *versus* .37,428 male). Over 40 % in all categories were male above 15 years of age. Women and children in migrant aggregates being studied were to be considered for malaria prevention and control apart from the men of working age group (>15 years age group). Pregnant women were highest in migrant groups from category 1 (23/94; 24.5 %) followed by category 2 (89/392; 22.7) and category 3 (225/1413; 15.9 %).

**Table 1:** Population structure by age and sex by category of migrant aggregates

Category	Under 5 years (male)	Under 5 years (female)	5-15 years (male)	5-15 years (female)	Above 15 years (male)	Above 15 years (female)	Total
Category 1 (*n* = 94)							
Sum	513 (5.77)	471	719	697	3838	2656	8894
%	5.77	5.29	8.08	7.84	43.15	29.86	100.0
Category 2 (*n* = 392)							
Sum	1522	1118	2362	1398	8206	5864	20470
%	7.44	5.46	11.54	6.83	40.09	28.65	100.0
Category 3 (*n* = 1,413)							
Sum	2213	2166	3015	2928	15040	11310	36672
%	6.03	5.91	8.22	7.98	41.01	30.84	100.0

Most of the migrant aggregates from category 3 reported Bago Region as their source of origin (category 1 = 63, category 2 = 230 and category 3 = 1,186). They came from nine townships being malaria endemic indicating the possibility of attaining the certain level of immunity. The reported number of transit places (sites between the source of origin and current work site) was few except those from category 3 (*n* = 149). The common transit was reported again as Bago Region across all categories. Migrant groups who reported their intention to move out from the current site was less than 10 % across three categories (9/94, 9.6 %; 14/392, 3.6 %; 140/1413, 9.9 %). Migrant groups in category 2 planned their next move towards larger geographical areas compared to categories 1 and 3. These areas covered Mon State, Ayeyarwaddy, Magway, Bago, Yangon, and Mandalay Regions including 15 townships mostly situated in malaria endemic areas and had a potential to spread drug-resistant malaria.

For category 1, gardening, construction, and farming were common (14.9, 27.7 and 16 % respectively). For category 2, most of the migrant aggregates engaged in rubber plantations and gold panning (36.7 and 26 % respectively). For category 3, major economic activity was farming (43.8 %). Migrant aggregates from category 2 reported nighttime work hours (84/392, 21.4 %) more than categories 1 and 3 due to the higher proportion of rubber plantation workers (144/392, 36.7 %; Table 2).

**Table 2:** Nature of work by category of migrant aggregates

Nature of work	Category1	Category2	Category3	Total
	*n* = 94	*n* = 392	*n* = 1,413	*n* = 1,899
Rubber	1 (1.1) (1.1)	144(36.7)	89(6.3)	234(12.3)
Gold panning	4 (4.1) (4.1)	108(27.5)	18(1.3)	130(6.8)
Bamboo cutting	2 (2.1) (2.1)	6(1.5)	116(8.2)	124(6.5)
Charcoal	5 (5.3) (5.3)	6(1.5)	152(10.8)	163(8.6)
Office staff	4 (4.3) (4.3)	3(0.8)	18(1.3)	25(1.3)
Gardening	14 (14.9) (14.9)	37(9.4)	44(3.1)	95(5.0)
Manual laborer	3 (3.2) (3.2)	12(3.1)	22(1.6)	37(1.9)
Dam works	7 (7.4) (7.4)	0(0.0)	5(0.4)	12(0.6)
Seller	1 (1.1) (1.1)	9(2.3)	35(2.5)	45(2.4)
Construction	26 (27.7) (27.7)	23(5.9)	29(2.1)	78(4.1)
Farming	15 (16.0) (16.0)	28(7.1)	619(43.8)	662(34.9)
Fishing	1(1.1) (1.1)	0(0.0)	198(14.0)	199(10.5)
Timber works	8(8.5)	3(0.8)	17(1.2)	28(1.5)
Missing	3 (3.2)	13(3.4)	51(3.6)	67(3.5)

Percentages are shown in parentheses

Around 38 % (139/370) of migrant aggregates in category 2 reported an easy access to rural health centers in contrast to migrant aggregates in category 1. Those aggregates were mostly accessible to rural health sub-centers (70.1 %; 61/87). However, compared to rural health centers, they were less accessible to station and township hospitals. For care provision, malaria volunteers were mostly within the reach of category 1 aggregates (43/66, 65.2 %) compared to category 2 (129/339, 38.1 %) and category 3 (619/1242, 49.8 %).

In the combined sample, most of the respondents had primary school level education (1004/2381, 42.2 %) and reported their stay in the area for less than six months (771/2381, 32.4 %). The median duration of stay was highest for respondents from category 1 (48 months) compared to other categories (12 months). Nearly 55 % of respondents did not report seasonal migration, mostly from category 2 (59.6 %). Only 26.6 % (634/2381) reported their intention to move out within one year and least by those respondents from category 1 (4/34; 11.8 %). Only 16 % of water supply in migrant aggregates was from tube wells. Sanitary latrines were most common in category 2 (478/862; 55.5 %). Around 64 % had 2–5 household members and the highest proportion was reported by respondents from category 2. Nearly 50 % of migrant households had one or more children under-five years of age. Most of them did not report any night time work within one week prior to the survey (2060/2381; 86.5 %).

Compared to those from category 1, the respondents from categories 2 and 3 were significantly less likely to receive some form of information, education and communication (IEC) related to malaria [20 % *vs*. 77 %; crude OR = 12.7(95 % CI −5.6-28.5) and 15 % *vs*. 77 %; crude OR = 18.8 (95 % CI- 8.4-43.0)]. Likewise, the respondents from categories 2 and 3 were significantly less likely to recall the visits of health staff to their places for malaria-related reasons such as seeking fever cases, testing with RDT, distribution of LLIN etc. compared to those from category 1 [31.4 % *vs*. 91.2 %; crude OR = 22.5 (95 % CI- 6.8-74.4) and 22.1 % *vs*. 91.2 %; crude OR = 36.5 (95 % CI- 11.1-119.9); Table 3].

**Table 3:** Information, attitudes and practices related to bed-nets by category of migrant group

Characteristic	Category 1	Category 2	Category 3	Combined
Received any form of IEC in malaria				
Yes	26(76.5)	176(20.4)	219(14.7)	711(25.3)
No	8(23.5)	651(75.5)	1,169(78.7)	1,947(69.4)
DK	0(0.0)	35(4.1)	97(6.5)	149(5.3)
Health staff visited for malaria related reasons				
Yes	31(91.2)	271(31.4)	328(22.1)	977(34.8)
No	3(8.8)	551(63.9)	1,071(72.1)	1,696(60.4)
DK	0(0.0)	40(4.6)	86(5.8)	134(4.8)
Number of treated bed nets at home				
Sum	27	707	767	2543
Mean ± SE	0.8 ± 1.1	0.9 ± 1.1	0.65 ± 0.77	1.1 ± 1.2
Range	0-4	0-8	0-6	0 -10
Willing to buy treated bed nets				
	*n* = 33	*n* = 755	*n* = 1,175	*n* = 1,963
	24 (72.7)	491 (65.0)	777 (66.1)	1292 (65.8)
One can’t catch malaria if sleeping regularly under treated bed net				
	*n* = 33	*n* = 756	*n* = 1,178	*n* = 1,967
Agree	31 (93.9)	682 (90.2)	965 (81.9)	1678 (85.3)
Disagree	1 (3.0)	27 (3.6)	35 (3.0)	63 (3.2)
Uncertain	0 (0.0)	37 (4.9)	129 (11.0)	166(8.4)
No response	1 (3.0)	10 (1.3)	49 (4.2)	60 (3.1)
Type of net slept last night ^a^				
	*n* = 34	*n* = 862	*n* = 1,485	*n* = 2,381
Ordinary	14 (41.2)	420 (48.7)	763 (51.4)	1197 (50.3)
ITN	1 (2.9)	119 (13.8)	132 (8.9)	252 (10.6)
LLIN	1 (2.9)	106 (12.3)	117 (7.9)	224 (9.4)
Number of family members slept under any type of net last night				
	*n* = 34	*n* = 862	*n* = 1,485	*n* = 2,381
None	6 (17.6)	66 (7.7)	81 (5.5)	153 (6.4)
One	5 (14.7)	145 (16.8)	295 (19.9)	445 (18.7)
2-4	7 (20.6)	389 (45.1)	529 (35.6)	925 (38.8)
≥5	16 (47.1)	262 (30.4)	580 (39.1)	858 (36.0)

Percentages shown in parentheses

^a^Total does not add up to 100 due to single item response

Most of the respondents from category 1 were fully aware of insecticide-treated bed nets (33/34, 97.1 %). But the awareness of long-lasting form was as low as 46.6 % (549/1167) as cited by respondents from category 2. As shown in Table 3, ownership of treated bed nets owned was reported to be as high as 767 in 1,485 households resided by 6,197 household members from respondents of category 3. Around 60 % of respondents across all categories cited that they had received ITN free-of-charge from the health department. Just over 70 % (24/33) of respondents from category 1 were willing to buy ITN slightly higher than the remaining categories. Around 30 % of respondents were aware of the availability of ITN at the town. Very few knew that ITN and LLIN required retreatment at six months after use and over 80 % lacked that particular knowledge. Less than 10 % were fully aware of the procedure to retreat the bed nets. Very high percentages across all categories agreed that *‘one can’t catch malaria if sleeping regularly under the treated bed net’*. In reality, the reported practice of sleeping under either the ordinary bed net or treated bed net was not high indicating the KAP gap. Likewise, in their households, only 38.8 % of respondents (925/2381) in the combined sample reported that at least 2–4 family members slept under any net, the night before the survey.

According to Table 4, their reported knowledge was high as regards the life-threatening nature of malaria, the high risk of contracting malaria by avoiding sleeping under treated bed-nets, and high risk of chance of catching malaria if working from dusk to dawn. As might be expected, fever and chills and rigor were widely recognized as common symptoms indicating malaria. However, only 59 % of respondents in the combined sample (highest in category 2 being 67.9 %) knew that malaria was due to the bite of mosquitoes carrying the malaria parasite. Very few knew Anopheles mosquitoes as the common vector in malaria transmission.

**Table 4:** Knowledge related to malaria by category of migrant group

Characteristic	Category 1	Category 2	Category 3	Total
Malaria is life threatening	*n* = 34	*n* = 862	*n* = 1,485	*n* = 2,381
Yes	33 (97.1)	807 (93.6)	1,314 (88.5)	2,154 (90.5)
No	0 (0.0)	15 (1.7)	24 (1.6)	39 (1.6)
Uncertain	1 (2.9)	29 (3.4)	109 (7.3) 0)	139 (5.8)
DK	0 (0.0)	11 (1.3)	38 (2.6)	49 (2.1)
If one doesn’t sleep under the treated net regularly, the chance to catch malaria is high				
Yes	33 (97.1)	794 (92.1)	1,212 (81.6)	2,039 (85.6)
No	1 (2.9)	19 (2.2)	47 (3.2)	67 (2.8)
Uncertain	0 (0.0)	39 (4.5)	174 (11.7)	213 (8.9)
DK	0 (0.0)	10 (1.2)	52 (3.5)	62 (2.6)
The chance to catch malaria is high, if working from dusk to dawn				
Yes	32 (94.1)	735 (85.3)	1,118 (75.3)	1,885 (79.2)
No	0 (0.0)	36 (4.2)	38 (2.6)	74 (3.1)
Uncertain	1 (2.9)	67 (7.8)	226 (15.2)	294 (12.3)
DK	1 (2.9)	24 (2.8)	103 (6.9)	128 (5.4)
Symptoms of malaria ^a^				
Fever	28 (82.4)	586 (68.0)	1,021 (68.8)	1,635 (68.7)
Chills & rigor	24 (70.6)	631 (73.2)	1,201 (80.9)	1,856 (78.0)
Sweating	7 (20.6)	182 (21.1)	461 (31.0)	650 (27.3)
Headache	23 (67.6)	411 (47.7)	785 (52.9)	1,219 (51.2)
Aches & pain	22 (64.7)	368 (42.7)	657 (44.2)	1,047 (44.0)
Malaria transmission ^a^				
Bitten by mosquito carrying malaria parasite				
	18 (52.9)	585 (67.9)	812 (54.7)	1,415 (59.4)
Bitten by anopheles mosquito carrying malaria parasite				
	0 (0.0)	95 (11.0)	173 (11.6)	268 (11.3)
Drink spring water	6 (17.6)	138 (16.0)	268 (18.0)	412 (17.3)
Closely attend malaria patient	1 (2.9)	83 (9.6)	117 (7.9)	201 (8.4)
Transfusion of infected blood	2 (5.9)	154 (17.9)	143 (9.6)	299 (12.6)

Percentages are shown in parentheses

*DK* Don’t know

^a^ Total does not add up to 100 due to single item response

Some 52.9 % of respondents from category 1 knew that one can prevent malaria by sleeping under the ordinary bed net. Conversely, respondents from category 2 knew more than the remaining categories that malaria was preventable by sleeping under the treated bed net (64 %; 549/862). Very few cited repellants as personal protection from malaria. Around 70 % of respondents from category 2 preferred to use treated bed nets to protect them from contracting malaria. Respondents accepted treated bed nets as an effective preventive measure for malaria but required strengthening (Table 5).

**Table 5:** Knowledge of prevention of malaria by category of migrant group

Characteristic	Category 1	Category 2	Category 3	Total
Prevention^a^	*n =* 34	*n* = 862	*n* = 1,485	*n* = 2,381
Slept under ordinary net	18(52.9)	319(37.0)	653(44.0)	990(41.6)
Slept under treated bed net	17(50.0)	549(63.7)	749(50.4)	1,315(55.2)
Repellants	5(14.7)	128(14.8)	152(10.2)	285(12.0)
Treated clothes	1(2.9)	70(8.1)	97(6.5)	168(7.1)
Treated hammock	0(0.0)	75(8.7)	84(5.7)	159(6.7)
Herbal decoctions	1(2.9)	48(5.6)	74(5.0)	123(5.2)
Indigenous medicine	2(5.9)	77(8.9)	96(6.5)	175(7.3)
Preferred measure ^a^				
Slept under ordinary net	14(41.2)	116(13.5)	263(17.7)	393(16.5)
Slept under treated net	18(52.9)	605(70.2)	973(65.5)	1,596(67.0)
Repellants	1(2.9)	13(1.5)	48(3.2)	62(2.6)
Treated clothes	0(0.0)	8(0.9)	26(1.8)	34(1.4)
Treated hammock	0(0.0)	14(1.6)	11(0.7)	25(1.0)
Herbal decoctions	0(0.0)	6(0.7)	16(1.1)	22(0.9)
Indigenous medicine	0(0.0)	20(2.3)	59(4.0)	79(3.3)

Percentages shown in parentheses

^a^ Total does not add up to 100 due to single item response

According to Table 6, the respondents knew RDT more than microscopy to confirm suspected malaria (29.4 % *vs*. 76.5 % in category 1; 20.1 % *vs.* 52.8 % in category 2; 21.5 % *vs*. 38 % in category 3) but differences were not significant. The results indicated their familiarity to rapid diagnosis in malaria mostly used by malaria volunteers and basic health staff (BHS) in study sites. Some 68.1 % of respondents from category 1 cited malaria volunteers as care providers for EDPT (P = 0.0005) whereas those from categories 2 and 3 were more familiar to public hospitals (P = 0.0001). Less than 20 % of respondents across all categories could cite ACT as their preferred antimalarial to cure the infection. However, approximately 36 % of respondents in the combined sample did not know this particular issue. Half of the respondents from category 1 received malaria-related information from volunteers, but 34.2 % of respondents from category 2 mentioned health staff as their major source of information. In category 3, around 24 % received information from their workmates and health staff.

**Table 6:** Knowledge related to early diagnosis and treatment of malaria by category of migrant group

Characteristic	Category 1	Category 2	Category 3	Total
	*n* = 34	*n* = 862	*n* = 1,485	*n* = 2,381
Microscopy				
Yes	10(29.4)	173(20.1)	319(21.5)	502(21.1)
No	1(2.9)	110(12.8)	45(3.0)	156(6.6)
DK	23(67.6)	579(67.2)	1,121(75.5)	1,723(72.4)
RDT				
Yes	26(76.5)	455(52.8)	564(38.0)	1,045(43.9)
No	0(0.0)	34(3.9)	15(1.0)	49(2.1)
DK	8(23.5)	373(43.3)	906(61.0)	1,287(54.1)
Personnel/facilities providing EDPT^a^				
Malaria volunteers	21(61.8)	155(18.0)	429(28.9)	605(25.4)
GP clinic	3(8.8)	139(16.1)	204(13.7)	346(14.5)
Worksite clinic	6(17.6)	35(4.1)	69(4.6)	110(4.6)
NGO clinic	1(2.9)	47(5.5)	48(3.2)	96(4.0)
Public hospital	9(26.5)	500(58.0)	852(57.4)	1,361(57.2)
RHC/subcenter	16(47.1)	448(52.0)	703(47.3)	1,167(49.0)
ACT				
Heard	12(35.3)	268(31.1)	428(28.8)	708(29.7)
Not heard	5(14.7)	131 (15.2)	320(21.5)	456(19.2)
DK	17(50.0)	463(53.7)	737(49.6)	1,217(51.1)
Preferred antimalarial that can cure infection				
ACT	6(17.6)	131(15.2)	183(12.3)	320(13.4)
Artesunate	5(14.7)	126(14.6)	310(20.9)	441(18.5)
Artemether	1(2.9)	20(2.3)	53(3.6)	74(3.1)
Quinine	1(2.9)	18(2.1)	71(4.8)	90(3.8)
Chloroquine	1(2.9)	32(3.7)	63(4.2)	96(4.0)
Analgesic	5(14.7)	144(16.7)	216 (14.5)	365(15.3)
Others^b^	0(0.0)	44(5.1)	43(2.9)	87(3.7)
DK	15(44.1)	347(40.2)	546(36.8)	908(38.1)

Percentages shown in parentheses

^a^Total does not add up to 100 due to single item response

^b^Others = Indigenous medicine packets and herbal decoctions

Table 7 analyzed attitudes related to diagnosis and treatment of malaria. Only 29 % of respondents from category 3 realized that irregular dose of antimalarials was harmful and less than 15 % recognized that artesunate monotherapy was undesirable. However, 77.6 % of respondents from category 2 realized that if someone with suspected malaria did not seek for EDPT within 24 h, there was a potential to suffer from severe malaria. Around 80 % of respondents from category 1 agreed that the head of the household was the most responsible person at home to facilitate the use of treated bed nets. And 76.5 % of respondents from the same category also agreed that heads of the household were mostly responsible for facilitating to seek for EDPT in acute undifferentiated fever.

**Table 7:** Attitudes related to diagnosis and treatment of malaria by category of migrant group

Characteristic	Category 1	Category 2	Category 3	Total
	*n* = 34	*n* = 862	*n* = 1,485	*n* = 2,381
Even the dose of antimalarials is irregular, nothing can happen				
Yes	10 (29.4)	246 (28.5)	351 (23.6)	607 (25.5)
No	12 (35.3)	383 (44.4)	436 (29.4)	831 (34.9)
DK	11 (32.3)	34 (24.8)	649 (43.7)	874 (36.7)
Uncertain	1 (2.9)	19 (2.2)	49 (3.3)	69 (2.9)
Trying Artesunate tablets alone do no harm (monotherapy)				
Yes	5 (14.7)	236 (27.4)	370 (24.9)	611 (25.7)
No	7 (20.6)	196 (22.7)	187 (12.6)	390 (16.4)
DK	17 (50.0)	385 (44.7)	766 (51.6)	1168 (49.1)
Uncertain	5 (14.7)	45 (5.2)	162 (10.9)	212 (8.9)
If someone with suspected malaria doesn’t seek for EDPT within 24 h, there is a potential to suffer from severe malaria				
Yes	21 (61.8)	669 (77.6)	826 (55.6)	1516 (63.7)
No	4 (11.8)	45 (5.2)	47 (3.2)	96 (4.0)
DK	5 (14.7)	124 (14.4)	486 (32.7)	615 (25.8)
Uncertain	4 (11.8)	24 (2.8)	126 (8.5)	154 (6.5)
Most responsible person at home to facilitate the use of treated bed nets				
Head	27 (79.4)	452 (52.4)	969 (65.3)	1448 (60.8)
Household member	6 (17.6)	325 (37.7)	334 (22.5)	665 (27.9)
Health Dept.	1 (2.9)	57 (6.6)	102 (6.9)	160 (6.7)
Volunteer	0 (0.0)	2 (0.2)	4 (0.3)	6 (0.3)
Employee	0 (0.0)	8 (0.9)	40 (2.7)	48 (2.0)
NGO	0 (0.0)	0 (0.0)	3 (0.2)	3 (0.1)
Missing	0 (0.0)	18 (2.1)	33 (2.2)	51 (2.1)
Most responsible person to facilitate seeking for EDPT in suspected malaria				
Head	26 (76.5)	460 (53.4)	921 (62.0)	1407 (59.1)
Household member	6 (17.6)	310 (36.0)	314 (21.1)	630 (26.5)
Health Dept.	1 (2.9)	60 (7.0)	176 (11.9)	237 (10.0)
Volunteer	0 (0.0)	3 (0.3)	3 (0.2)	6 (0.3)
Employee	0 (0.0)	16 (1.9)	40 (2.7)	56 (2.4)
NGO	0 (0.0)	0 (0.0)	1 (0.1)	1 (0.0)
Missing	1 (2.9)	13 (1.5)	30 (2.0)	44 (1.8)

Percentages shown in parentheses

Within past two weeks, few respondents in the combined sample reported acute undifferentiated fever (AUF) in adults (394/2381, 16.5 %) and children (191/1148, 16.6 %; Table 8). The AUF in this study referred to a self-reported fever that was of acute onset less than two weeks and could not be able to pinpoint the cause based upon previous experience among migrant groups. Around 32 % (11/34) from category 1, 22 % (185/862) from category 2 and 19 % (286/1485) from category 3 reported to seek help for AUF from nearby public facilities within past 6 months. However, the proportion that opted for the formal sector was approximately two to three times higher compared to general practitioners, NGO, volunteers and unlicensed practitioners (Table 8).

**Table 8:** Household experience of acute undifferentiated fever (AUF) and treatment-seeking practices for malaria by category of migrant group

Characteristic	Category 1	Category 2	Category 3	Total
Reported AUF in adults in past two weeks				
	*n* = 34	*n* = 862	*n* = 1,485	*n* = 2,381
Yes	3(8.8)	143(16.6)	248(16.7)	394(16.5)
No	30(88.2)	689(79.9)	1,153(77.6)	1,872(78.6)
DK^a^	1(2.9)	30(3.5)	84(5.7)	115(4.8)
Reported AUF in children in past two weeks				
	*n* = 16	*n* = 478	*n* = 654	*n* = 1,148
Yes	3(18.8)	73(15.3)	115(17.6)	191(16.6)
No	12(75.0)	366(76.6)	476(72.8)	854(74.4)
DK/NR^a^	1(6.2)	39(8.2)	63(9.6)	103(9.0)
Treatment seeking for AUF (past 6 months)				
	*n* = 34	*n* = 862	*n* = 1,485	*n* = 2,381
Public facilities	11(32.4)	185(21.5)	286(19.3)	482(20.2)
GP^b^/worksite clinic	6(17.6)	55(6.4)	85(5.7)	146(6.1)
NGO	6(17.6)	41(4.8)	126(8.5)	173(7.3)
Volunteers	8(23.5)	54(6.3)	140(9.4)	202(8.5)
Unlicensed practitioner	1(2.9)	43(5.0)	95(6.4)	139(5.8)

Percentages shown in parentheses

^a^DK = Don’t know

^b^GP = General Practitioner

## Discussion

Migration is a social determinant of health, and lack of migrant-specific health data may lead to gaps in policies, guidelines or programming [13]. In light of improved understanding of human movement by internal migrants as an important risk behavior, findings from this study might have an impact on malaria. In this study, the estimated total population over 55,000 was reported from nearly 1,900 migrant groups across 16 tier II townships in three states/regions of Myanmar. The proportion was highest in category 3 who were mostly at risk of malaria due to the lack of permanent work setting with implications for the local action plan for prevention and control of malaria. There is a possibility of malaria transmission on a spatial scale through movements of infected humans that exceed the limit of mosquito dispersal. The creation of local hot spots of malaria prevalence depends on interactions between the malaria parasites and its human and mosquito hosts [14, 15]. As noted by Smith and Whittaker [15], there is a need to develop collaborative community engagement efforts in regions of high population mobility.

Mobility dynamics of temporary migrant workers in this study is multifaceted depending upon various driving factors, especially economic opportunities. Among others, the mobility network is dominated by Bago Region, which forms a hub for human movements. This phenomenon is explained at least partly by the presence of agricultural opportunities and gold panning sites particularly common in tier II. This region thus attracts seasonal migrant workers from other parts of the country such as those from dry, arid zones. Likewise, one study from Brazil provided an evidence of mobility of non-immune people into endemic areas in light of the development of new rural economies such as plantations, rubber tapping industries, forestry work, etc. [16]. In this study, movements from other ecological zones (hilly region and delta) to the destination areas are less common compared to the dry zone. The evidence from areas targeted for malaria elimination has consistently focused on the role of human movements in introducing infections to areas targeted for malaria elimination. The occupational migration of human populations along the Cambodia-Thailand border is likely to contribute substantially to the spread of artemisinin resistance [17]. On the other hand, Tatem and Smith [18] highlighted the importance of natural migration boundaries that hampered the population mobility.

The reported source of origin of migrant aggregates in this study including transit sites were in malaria endemic regions of the country. Intended mobility to another site being expressed by migrant groups in the present study was not high, but their intended places for next movement especially from category 3 covered wider geographical area compared to remaining categories. The level of immunity to the local strain of parasites should be taken into account especially for mobile migrant populations [19]. However, malaria proneness depends on age and sex specific population structure. The presence of pregnant women seemed to be vulnerable to malaria in migrant groups and required proper antenatal care. Nearby health facilities well equipped for obstetric emergencies would be helpful to them. Moreover, living and working conditions of mobile migrant groups such as access to safe water and sanitation, nature of work, night time work might affect health. Likely factors for exposure to malaria risk across all categories included the presence of pregnant women and children under-five years of age, variable occupation such as working in rubber plantations (especially those in category 2) and had reported night time work with likely chances of being bitten by anopheles mosquitoes and poor access to health services especially public facilities (<40 %). Findings were also supported by evidence from the study in Thailand-Cambodia border among migrant workers [20].

Despite poor knowledge in malaria transmission, early diagnosis and adequate treatment and preventive measures, attitudes of mobile migrant workers towards an early diagnosis of acute undifferentiated fever suspected of malaria and treatment of malaria were satisfactory. Workmates and health staff could play an important role in migrant aggregates to channel the updated malaria information that required attention to program planners. However, the proportion seeking for help from the formal sector well equipped with RDT and antimalarials in support of MARC interventions was not high in the combined sample (482/2381;20.2 %; Table 8). Malaria causes only a small fraction of fever cases in malaria-endemic areas. In a rural hospital in India, among 1,197 adult patients being investigated for malaria, 88 % of them had a non-malarial acute undifferentiated fever (NMAUF). But many patients (39.9 %, 95 % CI 37.0–43.3) received antimalarial drugs [21]. One study in south-central Cambodia which assessed infectious etiologies of acute febrile illness among patients seeking health care identified only 7.5 % as malaria fever among 716 patients [22]. This issue calls for paying attention to NMAUF with empirical evidence in the treatment of such kind of fever with antimalarials [23, 24] either by self-medication or by health professionals. Further strengthening of EDPT services for malaria is essential in study sites to avoid progress into severity. Nevertheless, the effectiveness of disease interventions especially malaria was linked to population mobility. Evidence-based and comparable measures of vulnerability to malaria can be obtained from the specific sites with high levels of movement and their composition of sources and signs of malaria infection. In this connection, an earlier report elucidated that as heterogeneity in patterns of contact between pathogens and susceptible hosts, effective and efficient disease interventions should target the ‘nodes' of transmission [25]. In endemic countries including Myanmar, WHO recommended artemisinin combination therapies (ACTs) as the first-line treatment for uncomplicated *P. falciparum* malaria. The ACTs contain a short-acting artemisinin component that helps to reduce parasite burden and clinical symptoms quickly. Especially when provided together with a longer-acting partner drug, the mechanism to clear remaining parasites is highly effective [26]. Concerns of ready and easy access to ACTs should be addressed by finding constructive solutions in high-risk areas. This issue is important especially when faced with the availability of poor quality antimalarials in the locality [27]. Moreover, undocumented mobility can be captured through social network approaches and also through participatory development approaches that have already been proved as successful in the Thai-Cambodian border in Greater Mekong sub-region.

## Conclusions

Mobility dynamics of migrant groups was complex and characterized by circulatory as well as bidirectional movements in study townships. These groups had difficulty in accessing malaria-related information and in meeting malaria care providers except for more stable and permanent work settings. These aggregates were vulnerable to malaria especially in category 3 mostly linked to higher mobility patterns compared to categories 1 and 2. However, there was an opportunity for early diagnosis and prompt treatment by malaria care providers especially volunteers that required attention to improving refresher training on the regular basis, and to introduce mechanisms for prevention of attrition. Migrant households still required improved water and sanitation facilities so as to improve their general health conditions apart from malaria. Vulnerability to malaria may have been influenced by multiple factors. The associated factors included low level of education of migrant respondents, seasonal migration, short duration of stay, less contact with health services, lack of availability of malaria-related information, low rate of bed-net ownership especially treated bed-nets and their use, low level of knowledge of malaria vectors and bionomics, and poor knowledge of effective prevention. Migrants were familiar with rapid diagnostic tests for malaria mostly used by volunteers and basic health staff (BHS) in study sites but combined therapy for malaria needed wide recognition. Also, caution for non-malaria acute febrile illness in the context of rampant self-medication called for improved awareness. More importantly, re-designation of tier II townships is to be considered through the application of molecular approaches such as polymerase chain reaction thereby optimizing containment measures in these areas of high mobility.

## Additional file

Additional file 1:
**KAP of malaria among migrants working in malaria prone locations.** (PDF 195 kb)
