# Supplementary material for: Mobility dynamics of migrant workers and their socio-behavioral parameters related to malaria in Tier II, Artemisinin Resistance Containment Zone, Myanmar
Source: BMC Public Health. 2015 Sep 14;15:886. doi: 10.1186/s12889-015-2241-0 (PMC4570258; doi:10.1186/s12889-015-2241-0)
Supplement: Additional file 1: — KAP of malaria among migrants working in malaria prone locations. (PDF 195 kb) [file 12889_2015_2241_MOESM1_ESM.pdf]

KAP of malaria among migrants working in malaria prone locations

**State/Region:**<br>1=Kachin 2=Kayin 3= Bago 4= Kayah	**Township:---------------------------------**
**Type of cluster:** category= 1 category= 2	**Nearest village -----------------------------**
**Cluster ID :**	**Nearest health facility --------------------**
**Date of interview:**	**Start time:**

**SECTION A: Social and Demographic information**					
**SN**	**Question**				**Code**
1.	For how long do you stay in this area? (in months)				☐☐☐
2.	Do you migrate and work in this area seasonally?<br>YES =1 NO = 2 DK/NR =3				☐
3.	Do you have any intention to move out within one year?<br>YES =1 NO = 2 DK/NR =3				☐
4.	How many members are there in your household?				☐☐
5.	How many under five children are there in your household?<br>If none= fill in 99				☐☐
6.	How many members of your family work at night time in <u>past one week</u>?				☐☐
7.	Have you ever received any form of IEC related to malaria while you're staying in this area? YES =1 NO = 2 DK/NR =3				☐
8.	Does any type of health staff visit your household/worksite for malaria related reasons? YES =1 NO = 2 DK/NR =3				☐
9.	Have any of your adult household members ever experienced <u>acute undifferentiated fever</u> in <u>past two weeks</u>? **YES =1 NO = 2 DK/NR =3**				☐
10.	Have any children in your household ever experienced <u>acute undifferentiated fever</u> in <u>past two weeks</u>? **YES =1 NO = 2 DK/NR =3**				☐
11.	Have you/any of your household members ever sought treatment for malaria within <u>past 6 months</u>?				
		Yes	No	DK/NR	
11.1	Public hospital/RHC/Sub RHC	1	2	3	
11.2	GP clinic/company/work site clinic	1	2	3	
11.3	NGO clinic/malaria volunteer	1	2	3	
11.4	Community health worker/AMW	1	2	3	
11.5	Injectionist/unlicensed practitioner	1	2	3	
11.6	Others (specify)				
12.	What is the major source of water supply? **CIRCLE ONE ONLY**<br>stream=1 protected well=2 unprotected well= 3 river=4 rain= 5 pond/lake=6 others = 7 (specify)				☐
13.	What is the type of latrine used in your household? **CIRCLE ONE ONLY**<br>pit=1 flushed=2 surface=3 others=4 (specify)				☐
14.	How many bed nets (both treated & untreated) do you have at home? None=99				☐☐

15.	What is the highest level of formal schooling completed?<br>Illiterate/no formal schooling= 1 just read & write= 2 Primary=3 Middle=4 High=5 University/graduate=4				☐
	**SECTION B: Bed nets**				
16.	Have you ever heard of ITN? YES =1 NO = 2 DK/NR =3				☐
17.	Have you ever heard of LLIN? YES =1 NO = 2 DK/NR =3				☐
18.	How many LLIN/ITN do you have at home? None=99				☐☐
19.	Where did you last receive free LLIN/ITN? **CIRCLE ONE ONLY**<br>Never received free/bought=1 Employer=2 Health Department=3 NGO=4 Others=5 (specify)				☐
20.	Are you willing to buy LLIN/ITN? YES =1 NO = 2 DK/NR =3				☐
21.	Where can you buy LLIN/ITN?<br>**(CIRCLE MORE THAN ONE)**	**Yes**	**No**	**DK/NR**	
	1=village shop	1	2	3	
	2=town	1	2	3	
	3=work site	1	2	3	
	4=others (specify)	1	2	3	
22.	Did you sleep under bed net previous night?				
		Yes	No	DK/NR	
22.1	under ordinary bed-net	1	2	3	
22.2	under ITN	1	2	3	
22.3	under LLIN	1	2	3	
23.	How many household members sleep under any type of bed nets previous night? None= 99				☐☐
24.	When do you need to retreat ITN? (in months) DK/NR=99				------------
25.	When do you need to retreat LLIN? (in years) DK/NR=99				------------
26.	Are you aware of the procedure to impregnate bed nets?<br>YES =1 NO = 2 DK/NR =3				☐
27.	Sleeping under LLIN/ITN every night can prevent malaria.<br>1=agree; 2 =uncertain; 3=disagree; 4=DK/NR				☐
	**SECTION C: Malaria Information**				
28.	Malaria can be lethal. YES =1 NO = 2 Uncertain = 3 DK/NR =4				☐
29.	There is a high chance to catch malaria if one doesn't sleep under LLIN/ITN every night . YES =1 NO = 2 Uncertain = 3 DK/NR =4				☐
30.	There is a high chance to catch malaria during night time work (from dusk to dawn). YES =1 NO = 2 Uncertain = 3 DK/NR =4				☐
31.	Symptoms of malaria include:	**Yes**	**No**	**DK/NR**	
	31.1 Fever	1	2	3	
	31.2 Chills & rigor	1	2	3	
	31.3 Sweating	1	2	3	
	31.4 Headache	1	2	3	
	31.5 Muscle aches & pains	1	2	3	
	31.6 Others (specify)	1	2	3	

SN	Question				Code
32.	Malaria can be transmitted by	**Yes**	**No**	**DK/NR**	
	32.1 Bite of infected mosquito	1	2	3	
	32.2 Bite of infected anopheles mosquito	1	2	3	
	32.3 Drinking spring water	1	2	3	
	32.4 Transfusion of infected blood	1	2	3	
	32.5 Others (specify) --------------------	1	2	3	
33.	Malaria can be prevented by:	**Yes**	**No**	**DK/NR**	
	33.1 Sleeping under untreated bed net	1	2	3	
	33.2 Sleeping under LLIN/ITN	1	2	3	
	33.3 Using repellants	1	2	3	
	33.4 Herbal drinks	1	2	3	
	33.5 Indigenous medicine packet	1	2	3	
	33.6 Others (specify)---------------	1	2	3	
34.	What is your preference to prevent malaria? **CIRCLE ONE ONLY**<br><br>Sleeping under ordinary bed net = 1<br>Sleeping under LLIN/ITN = 2<br>Using repellants = 3<br>Herbal drinks = 4<br>Traditional medicine =5<br>Using impregnated clothes=6<br>Sleeping with impregnated hammock=7<br>Others (specify) ---------------------- = 8				☐
35.	How can you confirm fever suspected of malaria? **(UNPROMPTED)**<br>35.1 Microscopy YES =1 NO = 2 DK/NR =3<br>35.2 RDT YES =1 NO = 2 DK/NR =3				☐<br>☐
36.	Where can you confirm and get treatment for malaria? (place/person)	**Yes**	**No**	**DK/NR**	
	36.1 Volunteers	1	2	3	
	36.2 GP clinic	1	2	3	
	36.3 Company/work site clinic	1	2	3	
	36.4 Clinic from NGO	1	2	3	
	36.5 At public hospital	1	2	3	
	36.6 At RHC /sub RHC	1	2	3	
	36.7 Others (specify)	1	2	3	
37.	Have you ever heard of ACT?<br>YES =1 NO = 2 DK/NR =3				☐
38.	Which medicine will you prefer if you/your family suffer from malaria? **CIRCLE ONE ONLY**<br>1.ACT<br>2.Artesunate tablet<br>3.Artemether injection<br>4.Quinine<br>5.Chloroquine<br>6.Analgesics				☐
39.	If one doesn’t take full dose and complete course of malaria treatment, nothing serious can happen. YES =1 NO = 2 DK/NR =3				☐
40.	If one tries artemesinin tablets only for malaria nothing serious can happen.<br>YES =1 NO = 2 DK/NR =3				☐

SN	Question	Code
41.	There is a chance to suffer from severe malaria if a person with acute undifferentiated fever does not seek for diagnosis within 24 hours of onset. YES =1 NO = 2 DK/NR =3	☐
42.	Whom do you think is most responsible to use LLIN/ITN in the household? **CIRCLE ONE ONLY**<br>1. Head of the household<br>2. Myself<br>3. Health staff<br>4. Volunteers<br>5. Company staff<br>6. NGO	☐
43.	Whom do you think is most responsible to seek EDPT for acute undifferentiated fever? **CIRCLE ONE ONLY**<br>1. Head of the household<br>2. Myself<br>3. Health staff<br>4. Volunteers<br>5. Company staff<br>6. NGO	☐
44.	Where do you mostly receive any kind of information related to malaria? (symptom, diagnosis, treatment, prevention)<br>**CIRCLE TWO COMMONEST SOURCES**<br>1. Head of the household/family members<br>2. Work mates<br>3. Employers<br>4. Company doctor<br>5. Volunteers<br>6. Health staff<br>7. NGO<br>8. Others (specify)--------------	☐<br>☐

THANK YOU FOR YOUR CO-OPERATION

Name of interviewer	Signature
Name of supervisor	Signature
